# 

**Published:** 1859-05

**Authors:** 


NORTH AMERICAN

### MEDICO-CHIRURGICAL REVIEW.

Vol. III.] MAY, 1859. [No. 3

### CONTENTS.

### ANALYTICAL AND CRITICAL REVIEWS.

ART. PAGE

I.—The Transactions of the American Medical Association . . 385

II.—1. Observations on Diphtheritis. By W. F. Wade, M.D. . . 409

2. Diphtheritis: A Concise Historical and Critical Essay on the late Epidemic Pseudo-Membranous Sore-Throat of California, etc. By V. J. Fourgeaud, M.D. . . . . . 409

III.—The Ganglionic Nervous System; its Structure, Functions, and Diseases. By James George Davey, M.D. . . . . 419

IV.—Brief Expositions of Practical Medicine, to which is prefixed The Paradise of Doctors—a Fable. By Jacob Bigelow, M.D. 430

V.—On Animal and Vegetable Parasites of the Human Body; a Manual of their Natural History, Diagnosis, and Treatment. By Frederich Kuchenmeister, M.D. . . . . . 437

VI.—The Science and Art of Surgery; being a Treatise on Injuries, Diseases, and Operations. By John Erichsen, M.D. . 445

VII.—A Practical Treatise on the Diseases of Children. By D. Francis Condie, M.D. . . . . . . . 446

VIII.—The Physician's Pocket Day-Book, Visiting List, Diary, and Book of Engagements . . . . . . . . 446

### ORIGINAL COMMUNICATIONS.

I.—Contributions to Obstetric Jurisprudence. (No. 3.) By Horatio R. Storer, M.D. . . . . . . . . . 446

II.—The Present State of Microscopical Science, Medically Considered. By John H. Packard, M.D. . . . . . . 470

III.—Proceedings of the Pathological Society of Philadelphia . . 492

### AMERICAN PROGRESS OF MEDICAL SCIENCE.

Report on Practical Obstetrics for the Year 1858. By J. K. Mason, M.D. 501

### BI-MONTHLY FOREIGN ABSTRACTS

#### CHEMISTRY AND PHYSIOLOGY.

I.—On the Locality in which the Impressions of Taste are received . [illegible]

II.—Physiological Researches on Digestion . . . . . . [illegible]

III.—On a New Function of the Placenta and of the Amnios . . [illegible]

IV.—Observations on the Changes of the Urine in Disease . . . . 5[illegible]

PATHOLOGY AND THERAPEUTICS.

ART. PAGE
V.—Observations on Chorea Magna . . . . . . . . . 535
VI.—Observations on the Etiology and Treatment of Hemicrania . 536
VII.—Water-Glass, a Partial Substitute for Collodium . . . . 536
VIII.—Ethereal Oil of the Horse-Chestnut as a Topical Application in Gout and Rheumatism . . . . . . . . . 537
IX.—Ointment for Sore Nipples . . . . . . . . . . 537
X.—On the Diuretic Properties of Coffee . . . . . . . 538
XI.—On the Topical Use of Laudanum in Affections of the Eyes . 538

DISEASES OF WOMEN, AND OBSTETRICS.

XII.—Observations on the Use of the Tampon . . . . . 538
XIII.—Hæmatocele Retro-Uterina Catamenialis . . . . . . 539
XIV.—On the Treatment of Vesico-Vaginal Fistula . . . . . 540
XV.—On a Mechanical Impediment of Labor, not Sufficiently Noticed 540

SURGERY.

XVI.—New Method of Treating Powder-wounds . . . . . 541
XVII.—On the Treatment of Inflammation by Digital Compression . 541
XVIII.—On the Treatment of Burns by the Permanent Warm-Bath . 542
XIX.—Clinical Remarks on Dermoid Cysts . . . . . . . 542

BI-MONTHLY PERISCOPE.

SURGERY.

1. Upon the Causes of Death after Amputation . . . . . . 545
2. On Manual Compression in the Treatment of Aneurism and Inflammation . . . . . . . . . . . . . 549
3. A Contribution to the Statistics of Cancer, Collected from the Records of the Middlesex Hospital . . . . . . . . 551
4. Recent Strabismus Cured by Removal of Necrosed Bone from a Whitlow . . . . . . . . . . . . . . . 555

OBSTETRICS.

Report on the Cases of Ovariotomy that have occurred in Germany . 556

PRACTICE OF MEDICINE.

Retrospect on the Use of Raw Meat in the Diarrhœa of Weaned Children 559

THERAPEUTICS.

1. On the Use of Colchicum in Gout . . . . . . . . . 561
2. Glycerin in Chronic Laryngitis . . . . . . . . . 562
3. Glycerin in Hyperæsthesia Vulvæ, Erythema Nodosum, and Vaginitis 562

EDITORS' TABLE.—Hydrological Tour . . . . . . . . 563
Medical Students and Graduates in the United States during the Session of 1858–59.—Students in Philadelphia.—Professor Dickson's Introductory Lecture . . . . . . . . . . 568
The State Medical Society of Pennsylvania.—Philadelphia College of Pharmacy.—Pennsylvania College of Dental Surgery.—Pennsylvania Hospital.—New Medical School.—Albany Medical College.—Changes in Medical Schools . . . . . . . 569
University of the Pacific.—New Medical Journal.—American Veterinary Journal.—Acetated Tincture of Cimicifuga.—Discovery of the Circulation of the Blood.—Acknowledgments . . . . 570
[illegible]nsel's Salt . . . . . . . . . . . . . . 571
[illegible]GICAL NOTICES.—Dr. Mütter . . . . . . . . . 571
Boling . . . . . . . . . . . . . . . 574
G. M. Newton . . . . . . . . . . . . . 575
[illegible]. Alexander Monro . . . . . . . . . . . . 575
[illegible]NTHLY BIBLIOGRAPHICAL RECORD . . . . . . . . . 576

